# Supplementary material for: Native point defects of semiconducting layered Bi2O2Se
Source: Sci Rep. 2018 Jul 19;8:10920. doi: 10.1038/s41598-018-29385-8 (PMC6053437; doi:10.1038/s41598-018-29385-8)
Supplement: Supplementary file 1 — Supplementary Information [file 41598_2018_29385_MOESM1_ESM.pdf]

Supplementary Information

Native point defects of semiconducting layered Bi_2O_2Se

Huanglong Li[1]+, Xintong Xu[2+], Yi Zhang[3+], Roland Gillen[4], Luping Shi[1], John Robertson[5]*

[1] Department of Precision Instrument, CBICR, Tsinghua University, China, [2] School of Aerospace Engineering, Tsinghua University, China, [3] Department of Electronic Engineering, Tsinghua University, China, [4] Institute of Physics, Friedrich-Alexander-University of Erlangen-Nürnberg, Germany, [5] Engineering Department, University of Cambridge, UK

Corresponding author:
*Email: li_huanglong@mail.tsinghua.edu.cn

S1

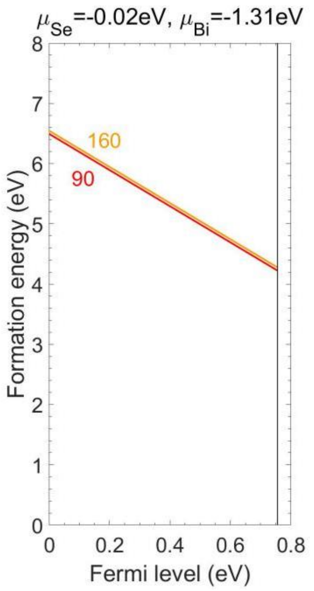

Figure S1 Formation energies of O_{Bi} in the highly negatively charged 3- state in the 90 and 160 atoms' supercells, for which the k meshes are 3x3x3 and 2x2x3, respectively.

S2

PDOSs (figure S2ab) of O_v^0 and Se_v^0 provide alternative way of understanding the shallow donor effect of O_v and Se_v, where we see that electrons are readily provided to the conduction band by thermal excitation at steady state. O_v^0 results in Bi dangling bonds which induce resonant defect states just above the CBM, whereas Se_v^0 renders no discernible defect state. The latter is attributed to the nonbonding characteristics of Se p band, reflected by the higher

energy position and much narrower bandwidth than that of O p band, in line with the weak electrostatic interaction between the Se plane and Bi_2O_2 layer.

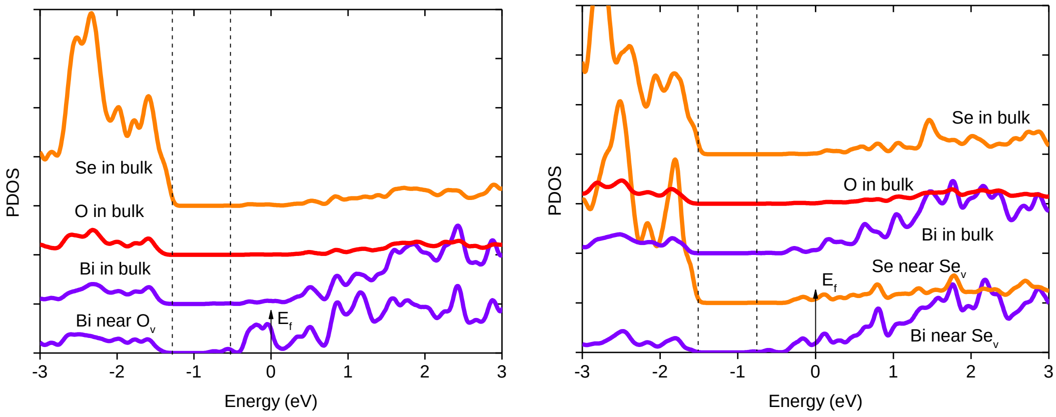

(a) (b)

Figure S2 PDOSs of charge neutral (a) O_v and (b) Se_v.

S3

PDOSs (figure S3) of Bi_v^0 provide alternative way of understanding the shallow acceptor effect of Bi_v, where we see that it is ready to accept electrons near the VBM by thermal excitation at steady state, leaving holes in the valence band.

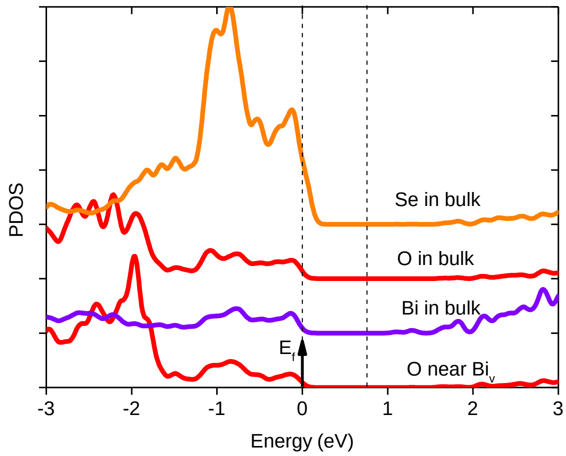

Figure S3 PDOSs of charge neutral Bi_v.
